# Revisiting hemoglobin constant spring: molecular insights, pathophysiological mechanisms, and clinical perspectives

**DOI:** 10.1186/s13023-025-04120-5

**Published:** 2025-11-25

**Authors:** Narawich Wongkhammul, Pinyaphat Khamphikham, Supawadee Maneekesorn, Pimlak Charoenkwan

**Affiliations:** 1 Department of Biochemistry, Faculty of Medicine, Chiang Mai University, Chiang Mai, Thailand; 2 Center of Multidisciplinary Technology for Advanced Medicine (CMUTEAM), Faculty of Medicine, Chiang Mai University, Chiang Mai, Thailand; 3 Division of Clinical Microscopy, Department of Medical Technology, Faculty of Associated Medical Sciences, Chiang Mai University, Chiang Mai, Thailand; 4 Hematology and Health Technology Research Center, Department of Medical Technology, Faculty of Associated Medical Sciences, Chiang Mai University, Chiang Mai, Thailand; 5 Division of Hematology and Oncology, Department of Pediatrics, Faculty of Medicine, Chiang Mai University, Chiang Mai, Thailand; 6 Thalassemia and Hematology Center, Faculty of Medicine, Chiang Mai University, Chiang Mai, Thailand

**Keywords:** Alpha-thalassemia, Gene therapy, *HBA2* gene, Hemoglobin constant spring, Genotype-phenotype correlation, Molecular basis, Termination codon mutation

## Abstract

**Background:**

Hemoglobin Constant Spring (Hb CS) is an α-globin variant predominantly found in Southeast Asian populations. Compound heterozygosity of Hb CS with α-thalassemia results in a spectrum of chronic hemolytic anemia. Recent advances in cellular therapy, particularly gene editing approaches currently under in-vitro investigation, offer potential new avenues for treatment in patients with severe phenotypes.

**Main body:**

This review discusses the molecular basis, pathophysiology, clinical manifestations, and current management strategies of Hb CS. The condition arises from a point mutation in the termination codon of the *HBA2* gene (*HBA2*:c.427T > C; p.Ter143Gln), leading to unstable mRNA, defective hemoglobin synthesis, and red blood cell membrane abnormalities. The clinical presentation of Hb H/Constant Spring disease and related genotypes ranges from mild anemia to severe hemolysis. In cases of homozygous Hb Constant Spring, affected fetuses may present with hydrops fetalis and severe intrauterine anemia; however, clinical improvement is typically observed after birth, with anemia often stabilizing to a milder form during infancy or early childhood. Management strategies vary from observation in mild cases to regular blood transfusions in more severe cases. Emerging gene editing techniques, including prime editing, represent promising therapeutic modalities under preclinical investigation.

**Conclusion:**

Hb CS is characterized by complex molecular mechanisms and a broad clinical spectrum. This review aims to provide a comprehensive overview to support improved clinical management of affected individuals and to explore future directions in therapeutic development.

**Supplementary Information:**

The online version contains supplementary material available at 10.1186/s13023-025-04120-5.

## Introduction

Hemoglobinopathies constitute a group of inherited blood disorders caused by mutations in the globin genes leading to structurally abnormal hemoglobin (Hb) or an impaired globin production resulting in thalassemia [1, 2]. Hb Constant Spring (Hb CS) is among the most common α-globin Hb variants in Southeast Asian populations [3, 4]. Hb CS can be coinherited with a two α-globin gene deletion on the other chromosome, resulting in Hb H/CS disease. The clinical presentation of Hb H/CS disease varies widely, ranging from mild to severe hemolytic anemia requiring lifelong transfusions or even resulting in hydrops fetalis [5–10]. This review aims to investigate the erythropoiesis process, pathophysiology, genotype-phenotype correlation, diagnosis, and treatment modalities in Hb H/CS disease, providing insights that could lay the foundational framework for the advancement of tailored therapy for the condition.

## α-globin gene clusters and the regulatory site

**Fig. 1:**
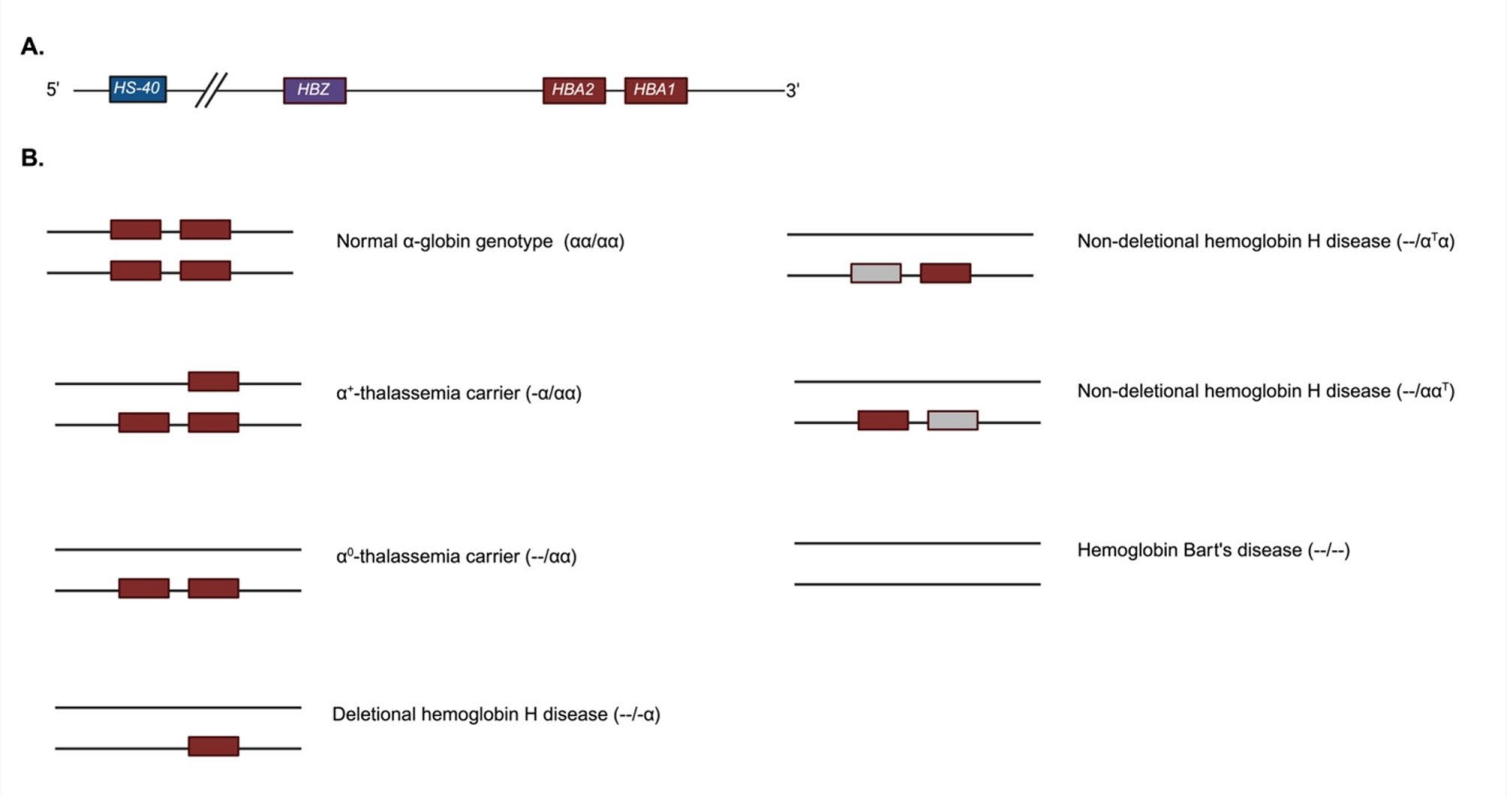
**(A)** Structure of the α-globin gene cluster **(B)** α-thalassemia genotypes. The brown boxes represent normal *HBA* genes. The grey boxes represent *HBA* genes with point mutation

Hb A, the major Hb in children and adults, is a tetramer consisting of two α-globin, two β-globin subunits, each with one heme molecule. α-globin is encoded by α-globin gene (*HBA*). Humans typically possess four functioning α-globin genes, two on each chromosome 16, known as *HBA2* and *HBA1.* The genes encoding α-globin and α-like globin protein are presented in a cluster 5’-*HBZ-HBA2-HBA1*-3’ as shown in Fig. 1A [11]. The *HBZ* gene encodes ζ-globin which is a component of embryonic Hbs, Hb Gower 1 (ζ_2_ε_2_), Hb Portland I (ζ_2_γ_2_) and Hb Portland II (ζ_2_β_2_). The *HBA2* and *HBA1* genes encode α-globin which is a component of fetal and adult Hbs, Hb F (α_2_γ_2_), Hb A (α_2_β_2_) and Hb A_2_ (α_2_δ_2_) [12]. Studies by Liebhaber et al. and Molchanova et al. reported higher mRNA expression of *HBA2* when compared to *HBA1*, with an *HBA2* to *HBA1* mRNA ratio at approximately 70:30 [13–15]. However, at the protein level, the α-globin production ratio is about 60:40, suggesting that *HBA2* mRNA is translated less efficiently than *HBA1* mRNA [14, 15]. The hypersensitive site-40 (HS-40) or known as multispecies conserved sequences-R2 (MCS-R2) is a regulatory site for the α-goblin gene cluster. It is located 40 kb upstream to the *HBZ* gene. The HS-40 region is important for chromatin looping and the binding of RNA Polymerase II at the promoters of α-globin genes [16–18]. 

## α-thalassemia and Hb variants resulting from mutations in the *HBA* genes

Thalassemia is inherited in an autosomal recessive mode. Figure 1B shows the genotypes of α-thalassemia. Most of the mutations in the *HBA* genes are large deletions affecting one or both α-globin genes. Individuals who harbor a deletion in one α-globin gene, are classified as α^+^-thalassemia (genotype -α/αα) carrier, which is known as silent carrier and those with a deletion of two α-globin genes on one chromosome are classified as α^0^-thalassemia (--/αα) carrier. The carriers are typically asymptomatic. Red blood cell (RBC) morphology of α^0^-thalassemia carriers may exhibit microcytosis, hypochromia, and mild poikilocytosis [15]. Non-deletional mutations or point mutations in one of the *HBA* genes (α^T^α/αα, αα^T^/αα) result in Hb variants with abnormal structure. The non-deletional mutations may also affect the production rate of α-globin gene [15, 19]. 

Hb H disease occurs when three out of the four functioning α-globin genes are affected leading to significantly reduced α-globin chain production. Hb H disease can be classified by the type of mutation to deletional Hb H disease (--/-α) and non-deletional Hb H disease (--/α^T^α, --/αα^T^). In Hb H disease, the reduction of α-globin chain production results in an accumulation of excess β-globin chains, forming abnormal Hb H molecules consisting of β-globin tetramer (β_4_) [15]. Patients with Hb H disease generally show manifestations of chronic extravascular hemolysis, and acute episodic intravascular hemolysis. Iron overload-related symptoms and chronic hemolysis are also common, particularly in adult patients, leading to conditions such as endocrinopathy and organ failure [20]. 

Hb Bart’s disease occurs when all four α-globin genes are deleted (--/--), resulting in the absent production of α-globin chains and a formation of Hb Bart’s, γ-globin tetramer (γ_4_), from the excess γ-globin chains. Patients with Hb Bart’s disease present with severe anemia from *in-utero.* Fetal anemia results in enlargement of liver and spleen and hydrops fetalis, characterized by accumulation of fluids in subcutaneous tissue, pericardial and peritoneal space. Patients with Hb Bart’s disease typically die *in-utero* or after birth [5, 20]. 

## Hb constant spring (Hb CS): molecular characteristics

α-globin Constant Spring variant (α^CS^) (*HBA2*:c.427T >C; p.Ter143Gln) arises from a nucleotide substitution of the termination codon, TAA by CAA, on the *HBA2* gene resulting in a substitution of termination codon by glutamine. This results in an abnormally long and unstable mRNA, and an abnormal α-globin chain with 31 additional amino acid residues to 172 amino acid residues [21]. These α^CS^-chains can combine with normal β-globin chains, leading to the formation of Hb CS molecules [19]. 

## The mRNA and transcriptomic characteristics

In a previous study α^CS^ mRNA levels were indirectly assessed in peripheral blood and bone marrow samples from individuals with Hb CS mutation using the α/β ratio determined through hybridization of RNA to cDNA. The findings revealed that α/β mRNA ratio in a patient with Hb H/CS disease was low, and the ratio was greater in bone marrow than in the peripheral blood [22]. Moreover, the α^CS^mutation causes ribosomes to read through the termination codon, resulting in translational extension into the 3’-UTR. This extended translation interferes with and disrupts the stable assembly of the α-complex, a protein structure that normally binds to the cytosine-rich stability determinants within the 3’-UTR. Without this protective complex, the α^CS^mRNA undergoes accelerated 3’ terminal deadenylation, leading to a significantly shorter poly(A) tail. Consequently, the abnormal α^CS^ mRNA has a significantly reduced half-life, leading to the α^+^-thalassemia-like effect due to the non-functioning *HBA2* gene [23]. The transcriptomic data of Hb H/CS erythroid cells also show increased expression of the heat shock protein genes (*HSP*s) as well as the chaperonin containing TCP-1 subunit genes (*CCT*s). These genes are involved in managing oxidative stress, preventing protein aggregation, and supporting erythroid differentiation [24]. The instability of Hb CS is also caused by an impaired interaction between the α^CS^ globin protein and α-Hb stabilizing protein (AHSP), a molecular chaperone that interacts with free α-Hb to facilitate the formation of a protein complex. This abnormality leads to excess of free α^CS^ globin which disrupts membrane protein stability [25, 26]. These factors collectively contribute to the pathophysiology and clinical manifestations observed in individuals with Hb CS mutation.

## Pathophysiology of Hb CS on RBC membrane

The pathophysiology of Hb CS involves multiple mechanisms affecting the RBC membrane. In deletional Hb H disease, excess β-globin chains form β-globin tetramers (Hb H), which aggregate into small, compact inclusion bodies. These Hb H inclusion bodies are typically retained within the RBCs and can lead to hemolysis [27]. In contrast, Hb CS inclusion bodies appear larger and more loosely organized, floating within the cytoplasm due to the loss of interaction of disulfide bonds between the precipitated material and the cell membrane. This loss of interaction is associated with the additional 31 amino acids in Hb CS, which lack cysteine residue [27]. 

A previous study demonstrated that RBCs and reticulocytes containing Hb CS exhibit a higher proportion of hypochromic cells compared to those with Hb H disease [28]. This phenomenon indicates the damage in the cell membrane and disruptions in volume regulation pathways [28]. The primary mechanism of membrane damage in Hb CS involves the direct association of oxidized α^CS^-globin chains with the RBC membrane and its skeleton structure. Unlike other forms of thalassemia, where damage is mainly caused by membrane-bound inclusion bodies, the unstable, oxidized α^CS^-globin chains directly disrupt membrane integrity. These interactions lead to structural and functional alterations, resulting in increased RBC membrane rigidity [27, 28]. 

The α^CS^-chain, which contains 14 hydrophobic amino acids, likely interacts with membrane transport sites, also leading to damage to the potassium-chloride (K-Cl) cotransporter which is responsible for regulating RBC volume [29, 30]. Dysregulation of the K-Cl cotransporter can lead to increased cellular hydration or swelling. This abnormal hydration of RBC containing Hb CS can further contribute to altered membrane properties, potentially decreasing the function and lifespan of the cells [28]. 

**Fig. 2:**
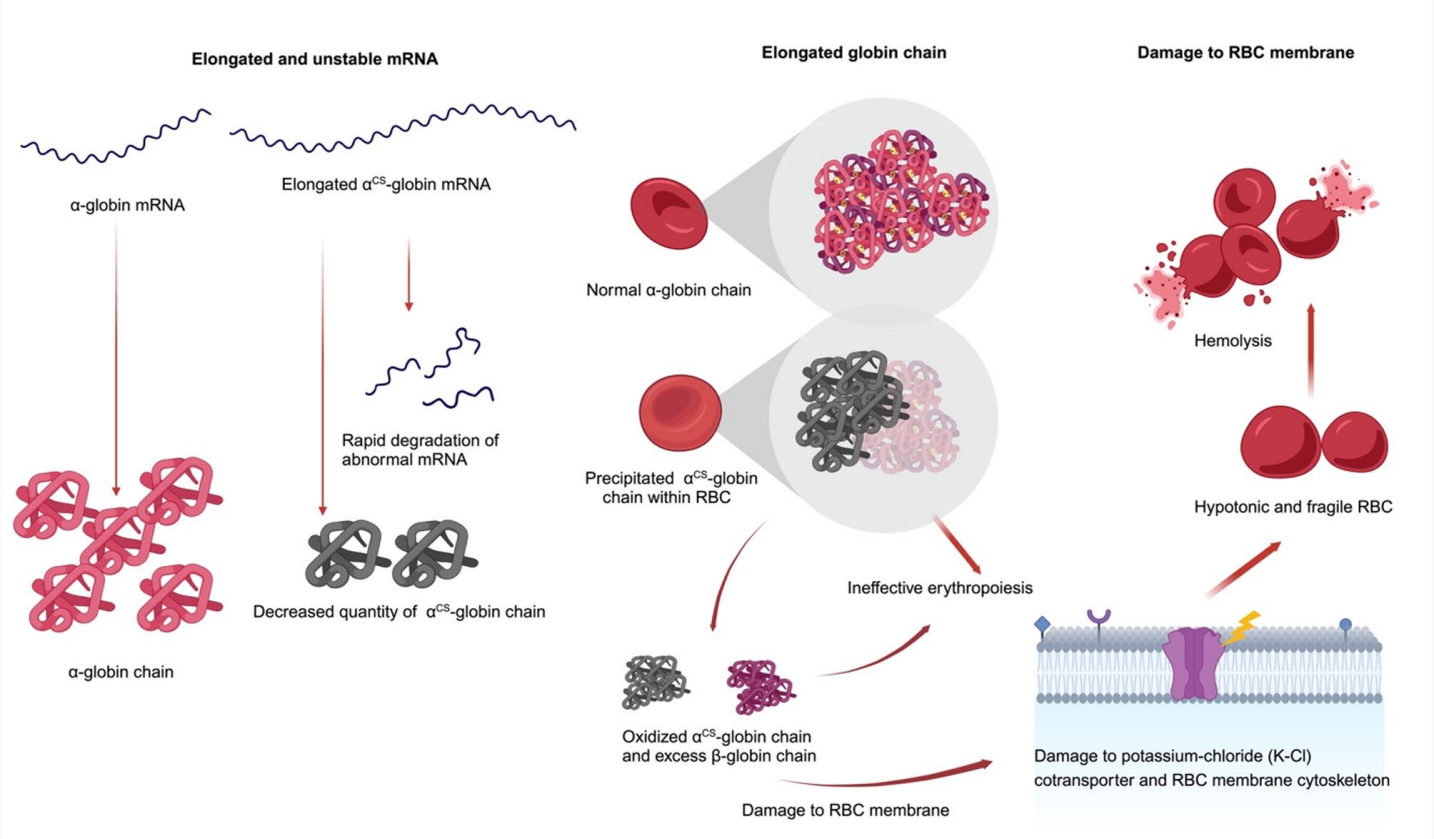
Pathophysiology of hemoglobin constant spring (CS) mutation at the levels of mRNA, globin protein, and red blood cell (RBC) membrane

A study on the number and maturation of reticulocytes in thalassemia demonstrated that patients with Hb H/CS disease and homozygous Hb CS exhibited delayed reticulocyte maturation compared to other type of thalassemia. Notably, Hb H/CS disease is associated with a high absolute reticulocyte count. These findings support the notion that Hb CS contributes to RBC damage. The delayed maturation is thought to result either from increased bone marrow release in response to enhanced RBC destruction or from a direct effect of Hb CS on erythroid maturation [31]. Furthermore, erythroid cells derived from patients with Hb H/CS disease exhibit a higher proliferation rate, reduced viability, and delayed terminal maturation compared to those from healthy controls, supporting the presence of ineffective erythropoiesis [24, 32]. These abnormalities contribute to both increased hemolysis and ineffective erythropoiesis in RBCs containing Hb CS, which may explain the greater clinical severity of Hb H/CS disease compared to deletional Hb H disease (Fig. 2).

## Epidemiology of Hb CS mutation

Hb CS was initially described by Clegg JB, et al. in 1971 in a family of Chinese descent from the city of Constant Spring in Jamaica [21]. Hb CS mutation is primarily found in Southeast Asia, particularly in Thailand, Laos, Cambodia, and Vietnam [4]. It is also prevalent in Southern China [33]. Although less common, sporadic reports of the Hb CS mutation have been documented in other regions, including the Mediterranean and the Middle East [34–36]. However, when examining the α^CS^ globin gene haplotype, differences in the origin of the Constant Spring mutation have been observed between Mediterranean and Southeast Asian populations [34]. 

The prevalence and allele frequency of Hb CS mutation in Southeast Asia as reported between 2010 and 2023 are shown in supplementary Table 1. The allele frequency of Hb CS varies from 0.0142 to 0.0775 [37–48]. 

## The clinical correlation and treatment

### Hb CS trait

Patients with the Hb CS trait (αα/α^CS^α) typically do not exhibit significant clinical symptoms and might only present with mild microcytic anemia, similar to the α-thalassemia trait [5]. 

### Hb H/CS disease

Hb H/CS (--/α^CS^α) results from the combination of a two α-globin gene deletion or α^0^ thalassemia with the α^CS^ mutation. Non-deletional mutations of the *HBA2* gene, such as the α^CS^ mutation, cannot be effectively compensated by an increase in *HBA1* gene expression. This results in less α-globin mRNA production from the remaining α-globin gene compared to deletional Hb H disease [13, 49]. Moreover, the disruption of RBC membrane, altered intracellular volume regulation, floating inclusion bodies, and impaired erythroid maturation can lead to more severe symptoms in Hb H/CS than in deletional Hb H disease. However, the clinical presentation of Hb H/CS can indeed vary widely, with some patients experiencing mild symptoms, while others might have more severe hemolytic anemia requiring long-term transfusion [5–8]. There have also been reports of fetal anemia and hydrops fetalis associated with Hb H/CS disease, also known as Hb H hydrops fetalis [9, 10]. The underlying mechanism behind this variability is still unclear and requires further research to be fully understood. Clinical variability is evident even among individuals with the same genotype, suggesting that additional genetic or environmental modifiers contribute to phenotypic differences [5]. Among these potential modifiers, one study demonstrated increased expression of alpha-hemoglobin-stabilizing protein (AHSP) in non-deletional Hb H disease, including Hb H/CS disease. Notably, higher AHSP expression levels were associated with greater disease severity, although AHSP genotypes and haplotypes did not account for this variability [50]. It has been postulated that AHSP stabilizes the limited pool of normal α-globin chains and offers some protection against the instability of α-globin variants, thereby enhancing Hb production. The highest AHSP expression was observed in the Hb CS and Hb Pakse group [50]. This finding is consistent with evidence from another study showing that both Hb CS and Hb Pakse have impaired binding affinity for AHSP [25]. Together, these findings suggest that upregulation of AHSP expression may act as a compensatory response and could influence disease severity in Hb H disease.

In addition to protein-level modifiers, recent research has identified significant alterations in m6A RNA methylation in Hb H/CS disease, leading to global mRNA hypomethylation [51]. This includes hypomethylation and reduced expression of the *BCL2A1* gene, which may play a role in erythroid differentiation [51]. These epitranscriptomic changes may represent an additional regulatory mechanism contributing to phenotypic variability. Beyond genetic and epigenetic factors, environmental influences such as nutritional status and recurrent infections are also likely to impact disease expression. Further studies are needed to elucidate the molecular mechanisms linking RNA methylation to disease severity and to better understand the multifactorial nature of phenotypic variability in Hb H/CS disease.

The treatment approach for Hb H/CS depends on the clinical severity of the individual patient. In some cases, patients may require occasional blood transfusions to manage their symptoms and maintain stable Hb levels. However, for more severe cases, regular and life-long blood transfusions may be necessary. Additionally, studies focusing on pregnancies involving individuals with Hb H/CS disease have indicated an elevated risk of adverse events during pregnancy. These complications include increased rates of preterm birth, low infant birth weight, and fetal growth restriction [52, 53]. 

### Homozygous Hb CS

Homozygous Hb CS (α^CS^α/α^CS^α) patients may exhibit varying clinical presentations depending on their age. Typically, individuals with homozygous Hb CS present with mild microcytic, hypochromic anemia [54]. There has been a report of intravascular hemolysis triggered by infection in a child with homozygous Hb CS [55]. Although generally considered as a mild condition, some cases may present with fetal anemia and hydrops fetalis [56–59]. Neonatal jaundice and anemia have also been reported [56]. These varying manifestations suggest that differences in the interactions between fetal Hb components or other *in-utero* environmental factors and Hb CS may contribute to the clinical variability.

### Diagnosis of Hb CS

The detection of Hb CS poses diagnostic challenges due to the small amount of α^CS^-globin chains produced. Initial screening typically relies on Hb analysis techniques such as capillary electrophoresis (CE) and high-performance liquid chromatography (HPLC). In patients with Hb H/CS disease, Hb H is usually detectable; however, the Hb CS peak may be small or entirely absent [60, 61]. For accurate diagnosis in suspected cases of Hb H/CS disease, molecular testing should be strongly recommended as part of standard diagnostic protocols. In individuals with Hb CS trait, hematological findings are often minimal or within normal ranges, making the detection of even a small Hb CS fraction an important indicator that should prompt molecular investigation.

Molecular diagnosis is the most reliable and definitive method for identifying Hb CS and differentiating it from other Hb variants. Allele-specific polymerase chain reaction (PCR), amplification refractory mutation system (ARMS), and PCR-high-resolution melting (HRM) analysis are common techniques used to detect Hb CS mutation [62–66]. Dot blot hybridization and PCR-RFLP have also been widely employed [67–69]. Sequencing techniques such as Sanger sequencing, next-generation sequencing and third generation sequencing platforms remain the most reliable approach to unambiguously identifying Hb CS [70, 71]. 

## Novel treatment: gene editing

The conventional treatment for severe cases of Hb H/CS disease, involves RBC transfusions and iron chelation therapy [20]. Allogeneic hematopoietic stem cell transplantation is a recommended curative treatment option for patients with severe thalassemia who have donors with compatible HLA [1, 20]. 

Gene editing is a novel treatment approach for Hb H/CS disease, with CRISPR/Cas9 (clustered regularly interspaced short palindromic repeats and CRISPR-associated protein 9) being one of the most versatile tools for therapeutic applications. This genome editing technology has been utilized to alter targeted genes in several clinical studies [72–74]. The CRISPR/cas9 system can precisely target specific DNA sequences and induce double-stranded breaks, thereby activating DNA repair pathways such as non-homologous end joining (NHEJ) and homology-directed repair (HDR). Through these repair pathways, researchers aim to correct mutations using different strategies, such as inducing insertions or deletions (indels) in the target gene via NHEJ or correcting mutations through HDR [75, 76]. 

CRISPR/Cas9-mediated correction of the Hb CS mutation has been successfully performed in vitro using induced pluripotent stem cells (iPSCs) and fibroblast cells [77, 78]. However, this approach has limitations, as the double-stranded DNA breaks can lead to high cellular toxicity and trigger apoptosis through activation of the p53 pathway, as well as cause genome rearrangements. Moreover, improper activation of double-stranded breaks repair pathways has been linked to developmental defects, neurological disorders, and an increased risk of cancer [76, 79, 80]. Another limitation is that HDR, the precise repair mechanism required for accurate gene correction, is restricted to the G2 and S phases of the cell cycle, resulting in low editing efficiency [81]. 

Base editing and prime editing are novel genome-editing techniques developed as improvements over the traditional CRISPR/Cas9 system [82, 83]. Base editing provides a safer and more efficient alternative to traditional CRISPR/Cas9 method by enabling precise single-base conversions such as cytosine-to-thymine (C >T) conversions using cytosine base editors and adenine-to-guanine (A >G) conversions using adenine base editors, without inducing double-stranded DNA breaks or requiring donor DNA templates. This approach significantly reduces the risk of indels [82, 84]. On the other hand, prime editing provides an even broader editing spectrum, including all 12 possible single base conversions, small insertions, and deletions. This system offers flexible editing capabilities that surpass those of earlier genome-editing methods [83, 85, 86]. The Hb CS mutation represents a promising target for both base editing and prime editing. Correcting this mutation could potentially convert individual with Hb H/CS disease (--/α^CS^α) into an α-thalassemia trait genotype (--/αα), thereby significantly reducing disease severity. Recently, Congwen et al. successfully applied prime editing to correct the Hb CS mutation in patient-derived hematopoietic stem cells. However, a limitation of the study is that the long-term editing efficiency in vivo remains unassessed due to an insufficient number of edited cells for transplantation experiments [87]. Further in vivo and ex vivo studies are needed before this approach can be applied clinically to patients with Hb H/CS disease. Nevertheless, these advancements highlight the therapeutic potential of gene editing for treating Hb H/CS.

## Other termination codon mutations of the *HBA2gene*

**Fig. 3:**
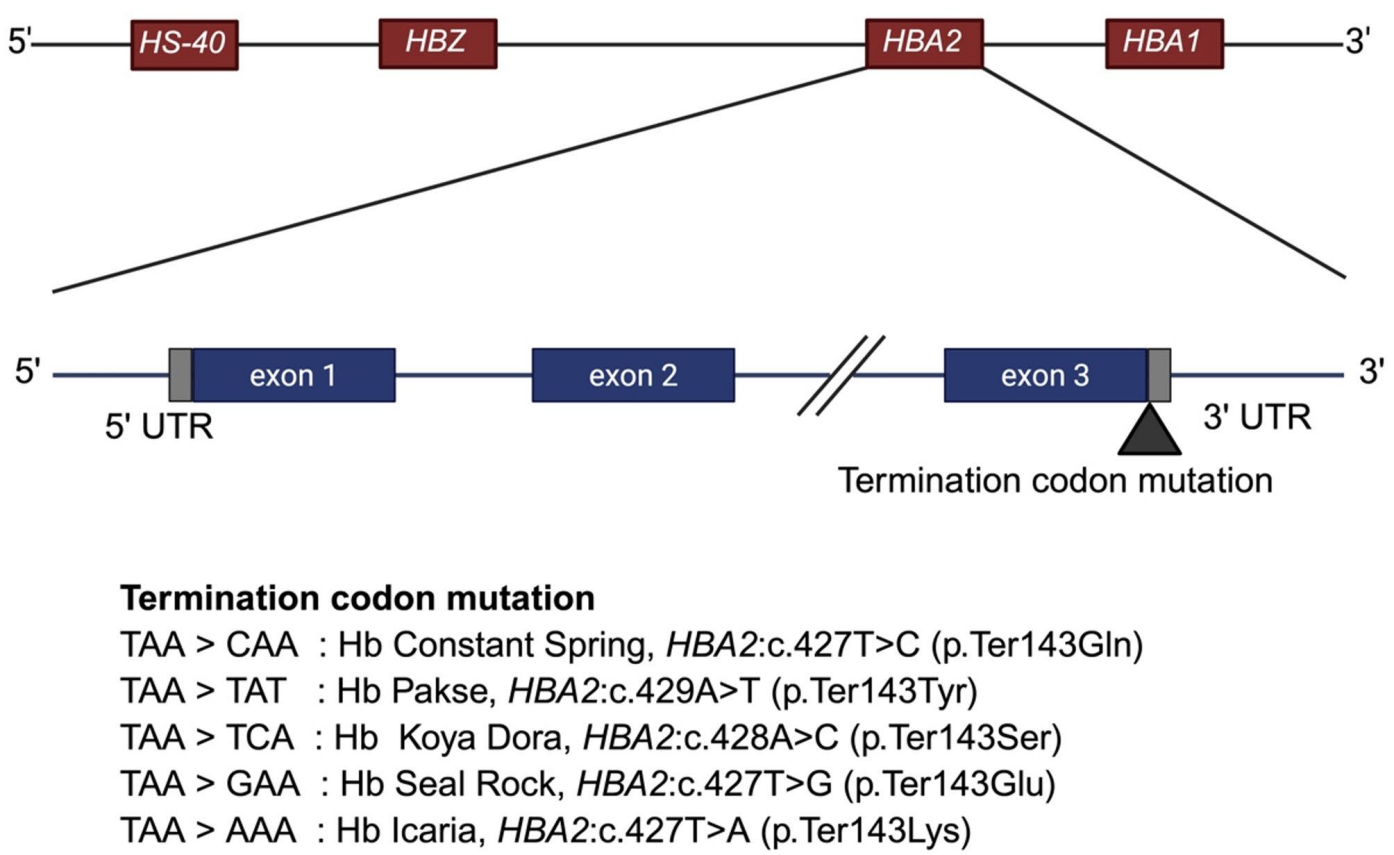
Mutations in the *HBA2* termination codon

Other than Hb CS mutation, four additional mutations at the termination codon of the *HBA2* gene have been identified: Hb Pakse [88, 89], Hb Koya Dora [90, 91], Hb Seal Rock [92] and Hb Icaria [93–96]. All of these mutations result in the substitution of the termination codon with an amino acid, leading to an extension of 31 amino acids. These variants are illustrated in Fig. 3. The clinical manifestations and hemoglobin electrophoretic findings of Hb H/CS disease and Hb H/Pakse disease are largely similar [89]. A recent case series involving individuals with compound heterozygosity for Hb Koya Dora and a 3.7 kb α-globin gene deletion, as well as those homozygous Hb Koya Dora, demonstrated comparable clinical and hematologic profiles to patients with Hb CS [91]. Similar findings have also been reported in a rare case of Hb Seal Rock in combination with a 3.7 kb deletion [92]. Likewise, patients with Hb H/Hb Icaria exhibit overlapping clinical and laboratory features [95, 96]. 

## Conclusions

Hb CS is clinically significant due to its association with a variable phenotype that can include severe hemolytic anemia. In Hb H/CS disease, clinical severity ranges from non-transfusion-dependent anemia to severe, transfusion-dependent anemia. Additionally, homozygous Hb CS can present with severe fetal anemia or hydrops fetalis. This phenotypic variability highlights the importance of accurate diagnosis, genetic counseling, close clinical monitoring, and appropriate transfusion support in severe cases. Emerging therapeutic strategies, including gene editing, hold promise for reducing transfusion needs and improving long-term outcomes. Continued efforts in early detection and the development of targeted therapies are essential to alleviating the clinical burden associated with this Hb variant.

## Electronic Supplementary Material

Below is the link to the electronic supplementary material.

Supplementary Material 1

## Data Availability

Not applicable.

## Abbreviations

3’UTR: 3’ untranslated region
AHSP: α-Hb stabilizing protein
ARMS: Amplification refractory mutation system
CCTs: Chaperonin containing TCP-1 subunit genes
CE: Capillary electrophoresis
CRISPR/Cas9: Clustered regularly interspaced short palindromic repeats and CRISPR-associated proteins 9
Hb: Hemoglobin
Hb CS: Hemoglobin Constant Spring
HBA: α-globin gene
HDR: Homology-directed repair
HPLC: High performance liquid chromatography
HRM: High-resolution melting
HSPs: Heat shock proteins genes
HS-40: The hypersensitive site-40
iPSCs: Induced pluripotent stem cells
K-Cl: Potassium-Chloride
MCS-R2: Multispecies conserved sequences-R2
NHEJ: Non-homologous end joining
PCR: Polymerase chain reaction
RBC: Red blood cell
