# Supplementary material for: Revisiting hemoglobin constant spring: molecular insights, pathophysiological mechanisms, and clinical perspectives
Source: Orphanet J Rare Dis. 2025 Nov 25;20:640. doi: 10.1186/s13023-025-04120-5 (PMC12750903; doi:10.1186/s13023-025-04120-5)
Supplement: Supplementary file 1 — Supplementary Material 1 [file 13023_2025_4120_MOESM1_ESM.docx]

**Supplementary Table 1**: The prevalence and allele frequency of Hb Constant Spring mutation in Southeast Asia

| **Genotype** | **Prevalence (%)** | **Allele frequency of Hb CS** | **Population** | **References** | **Country** |
| --- | --- | --- | --- | --- | --- |
| αα/α^CS^α   1. -α^3.7^/α^CS^α 2. --^SEA^/α^CS^α 3. β/β^E^, αα/α^CS^α 4. β/β^E^, -α^3.7^/α^CS^α   β/β^E^, --^SEA^/α^CS^α | 2.49  0.18  0.36  0.89  0.18  0.18 | 0.0214 | 566 newborns | Charoenkwan P, et al 2010 (37) | Thailand |
| αα/α^CS^α   1. -α^3.7^/α^CS^α 2. --^SEA^/α^CS^α   α^CS^α/α^CS^α | 4.42  0.51  0.51  0.17 | 0.0289 | 512 newborns | Pichanun D, et al 2010 (38) | Thailand |
| αα/α^CS^α  α^CS^α/α^CS^α   1. -α^3.7^/α^CS^α 2. β/β^E^, αα/α^CS^α   β/β^E^, -α^3.7^/α^CS^α | 2.1  0.5  0.5  2.1  0.5 | 0.031 | 190 children | Panomai N, et al 2010 (39) | Thailand |
| αα/α^CS^α  α^CS^α/α^CS^α   1. -α^3.7^/α^CS^α 2. --/α^CS^α   α^PS^α/α^CS^α | 2.31  0.55  0.77  0.07  0.07 | 0.0216 | 1431 individuals | O'Riordan S, et al 2010 (40) | Vietnam |
| αα/α^CS^α   1. -α/α^CS^α 2. --/α^CS^α   β/β^E^, αα/α^CS^α   1. β/β^E^, -α^3.7^/α^CS^α 2. β/β^E^, α^PS^α/α^CS^α 3. β/β^E^, α^CS^α/α^CS^α   β^E^/β^E^, αα/α^CS^α | 7.01  0.88  0.44  3.09  0.88  0.44  0.44  0.44 | 0.0703 | 226 newborns | Srivorakun H, et al 2011 (41) | Thailand |
| α^CS^α/ | 10.6 | 0.053 | 715 pregnant women | Tritipsombut J, et al 2012 (42) | Thailand |
| αα/α^CS^α   1. -α^3.7^/α^CS^α 2. --/α^CS^α 3. β/β^E^, αα/α^CS^α   β/β^E^, -α^3.7^/α^CS^α | 3.7  0.83  0.41  2.49  1.24 | 0.04335 | 241 newborns | Uaprasert N, et al 2013 (43) | Thailand |
| αα/α^CS^α  α^CS^α/α^CS^α   1. -α/α^CS^α 2. β/β^E^, αα/α^CS^α   β/β^0^, αα/α^CS^α | 20.5  2.4  0.67  2.4  0.34 | 0.143 | 298 individuals | Nguyen VH, et al 2014 (44) | Vietnam |
| αα/α^CS^α  α^CS^α/α^CS^α  -α^3.7^/α^CS^α | 10.2  1.2  2.9 | 0.0775 | 578 pregnant women | Wisedpanichkij R, et al 2015. (45) | Thailand |
| α^CS^α/ | 2.84 | 0.0142 | eight ethnic groups | Lithanatudom P, et al 2016 (46) | Thailand |
| αα/α^CS^α | 4.23 | 0.02115 | 638 pregnant women | Pharephan S, et al 2016 (47) | Thailand |
| 1. --/α^CS^α | 21.4 | - | 28 patients with α-thalassemia disease | Yatim NF, et al 2014 (48) | Malaysia |

Abbreviations: CS, Constant Spring; Hb, hemoglobin; PS, Pakse
